# Peritoneal Fluid Modulates Redox Balance and RNA Integrity in Mouse Oocytes: Insights into Endometriosis-Related Oxidative Stress

**DOI:** 10.3390/antiox14081018

**Published:** 2025-08-20

**Authors:** Joanne Horton, Simon Lane, Ying Cheong

**Affiliations:** 1 Human Development and Health, Faculty of Medicine, University of Southampton, Southampton SO16 6YD, UK; joanne.horton@uhs.nhs.uk; 2 Department of Chemistry, University of Southampton, Southampton SO17 1BJ, UK; simon.lane@soton.ac.uk

**Keywords:** antioxidant, reactive oxygen species (ROS), endometriosis, fertility, reproduction, oocytes, peritoneal fluid

## Abstract

Reactive oxygen species (ROS) are vital for oocyte development, yet the redox state of peritoneal fluid may differ between health and disease. This study investigates the effects of peritoneal fluid from women with and without endometriosis on mouse oocytes’ redox status and RNA oxidation. Peritoneal fluid samples were collected during laparoscopy from women enrolled in an ethically approved case–control study. Stimulated C57BL6 mouse germinal vesicle oocytes were microinjected with RNA transcribed from a Grx1-roGFP2 construct and imaged to assess redox changes. Further oocytes were incubated in standard media, H_2_O_2_, or 20% peritoneal fluid, fixed, and immunostained for 8-OHG to evaluate RNA oxidative damage. Oocytes exposed to endometriosis-affected peritoneal fluid showed significantly less redox reduction (mean change 0.07, *p* < 0.001) compared to fluid from unaffected women (mean change 0.17, *p* < 0.001), suggesting impaired antioxidant capacity. Those treated with fluid from women without endometriosis showed a more significant reduction (mean ratio change 0.17, *p* < 0.001). RNA damage was higher in oocytes incubated in fluid from women with infertility compared to pelvic pain (*p* < 0.001). These findings suggest an altered oxidative environment of peritoneal fluid in endometriosis may contribute to impaired oocyte quality, highlighting a potential mechanism of infertility in affected women.

## 1. Introduction

Reactive oxygen species (ROS) are a number of reactive molecules or “free radicals” mostly generated as a by-product of mitochondrial oxidative phosphorylation where they are released during the mitochondrial electron transport of aerobic metabolism to generate energy in the form of ATP [1]. ROS and its closely related reactive nitrogen species can also be formed by catalytic/oxidative enzymes (e.g., NADPH oxidase, lipoxygenases and peroxisomes) and metal cations (e.g., copper and iron) in the body [2]. As well as these endogenous sources, ROS is also produced by some exogenous sources; oxidising drugs (e.g., chemotherapeutics), environmental toxins (e.g., bleach, smoking) and ionising radiation (e.g., UV or gamma radiation) [3]. Inflammatory conditions lead to a higher level of ROS production due to its effect on mitochondria and oxidative enzymes. Oxidative stress occurs when there is an imbalance towards the pro-oxidative state either by increase in ROS or by reduction in antioxidant defence mechanism. Oxidative stress results in direct or indirect damage of proteins, lipids and nucleic acids and in this way, contributes to the pathogenesis of many diseases.

Endometriosis is a leading cause of infertility, and oxidative stress has been identified as a key driver of oocyte damage and impaired reproductive potential in affected women [4,5,6]. Oxidative stress markers are elevated in endometriosis patients in serum, urine, peritoneal fluid, follicular fluid, ovarian cortex and endometrial tissue (ectopic and eutopic) [5,7]. Increased peritoneal production of reactive oxygen species (ROS) via enhanced macrophage degradation of erythrocytes—with consequent excess release of heme and iron—may underlie the elevated ROS levels observed in endometriosis [7,8,9,10]. In follicular fluid, ROS is theorised to damage the oocyte’s cytoskeleton, cell membrane, and DNA, impairing oocyte development and quality and contributing to the fertility deficits seen in endometriosis [7,11]. Both oocyte and sperm DNA damage has also been reported following exposure to the peritoneal fluid of women with endometriosis [12,13].

Peritoneal fluid constitutes the microenvironment for key reproductive events, including ovulation, oocyte pick-up, and fertilisation. It represents a significant component of the fluid within the fallopian tubes where early embryogenesis occurs [14]. In endometriosis, this fluid is in direct contact with endometriotic lesions and contains increased levels of ROS, growth factors, cytokines, and various immune cells [15]. Despite extensive characterisation of oxidative stress markers in endometriosis, the direct effects of peritoneal fluid on oocyte intracellular redox balance and RNA integrity remain poorly understood. This gap is particularly critical since the intrinsic buffering of the redox environment may dictate how oocytes respond to ROS exposure.

It remains unclear whether elevated ROS directly affects the oocyte’s intracellular redox status and RNA integrity. Fully grown germinal vesicle (GV) oocytes are transcriptionally quiescent and depend solely on pre-stored mRNAs for protein synthesis until zygotic genome activation [16].

Consequently, the GV-stage oocyte and early embryo may be particularly vulnerable to RNA damage. We hypothesise that higher levels of ROS in peritoneal fluid from women with endometriosis will alter the redox status within oocytes. We hypothesise that consequently, increased levels of RNA damage occur within the oocyte when exposed to peritoneal fluid of women with endometriosis compared to women without endometriosis. To investigate this, mouse oocytes will be exposed to peritoneal fluid from women with and without endometriosis, with subsequent evaluation of intracellular redox status using an intracellular biosensor and assessment of RNA damage via immunofluorescence with anti-8-OHG.

## 2. Materials and Methods

### 2.1. Study Population

This study utilised a case–control design to compare the effects of peritoneal fluid from women with (cases) and without (controls) endometriosis on mouse oocytes. The study used peritoneal fluid samples from women recruited through two ethically approved studies: Peritoneal Fluid Biology in Health and Disease and XSESS: Oxidative Stress in Women with Endometriosis Pre- and Post-Surgery.

Peritoneal fluid biology in health and disease was a previously approved study commenced in 2008 and was used to begin sample collection. For use in this work the study was re-registered with the current Integrated Research Application System (IRAS) and two amendments were approved by the national ethics committee and the Faculty of Medicine ethics committee of the University of Southampton. All patients gave informed written consent to take part in the study. XSESS: oXidative Stress in women with Endometriosis pre and poSt Surgery was set up and met ethical approval with a national ethics committee, the Faculty of Medicine ethics committee of the University of Southampton and was sponsored by the Research and Development Department of Southampton General Hospital. The study was powered for the primary outcome of comparing reactive oxygen species levels in the peritoneal fluid, serum and urine of women with and without endometriosis before and after laparoscopic surgery. 62 patients were recruited. 12 of these participants’ peritoneal fluid samples have been used for the secondary aim of the study, to investigate the impact on oocyte RNA. Participants included women aged ≥ 18 years (age of an adult in the NHS) who were undergoing laparoscopic surgery for suspected or confirmed endometriosis or tubal sterilisation (to facilitate equal recruitment of patients with endometriosis and healthy controls) at Princess Anne Hospital, Southampton, a tertiary referral centre for endometriosis management. Exclusion criteria included menopause, pregnancy or active infections.

Eligible participants were recruited from the gynaecology department at Princess Anne Hospital. Screening was performed and women fulfilling the inclusion/exclusion criteria were sent an information leaflet and letter of invitation prior to their surgery. Those women were approached on the day of their surgery and if interested in taking part, were able to discuss the study. Women who decided to participate completed a consent form and were asked questions pertaining to medical history and to complete the WERF-EPHect (World Endometriosis Research Foundation Endometriosis Phenome and biobanking harmonisation project) study proforma. Participant information regarding factors that may affect ROS levels such as co-morbidity, age and smoking status were therefore obtained. For the XSESS study these consenting women also completed a pelvic pain questionnaire. Recruited participants then underwent their surgical treatment as planned and the WERF-EPHect Minimal Surgical Form was completed to document any pelvic pathology, including the American Society of Reproductive Medicine scoring of endometriosis. Participants attended a post-operative follow up appointment with their clinical team, at which time they repeated a pelvic pain questionnaire and had a blood sample and urine sample taken for research.

### 2.2. Sample Collection

Peritoneal fluid was aspirated intraoperatively using either a Wallace Embryo Replacement Catheter or a 14-gauge Ryles tube, and centrifuged before storage at −80 °C. Processing parameters differed between studies:

Peritoneal Fluid Biology Study: Transfer to laboratory on wet ice followed by centrifugation at 900× *g* for 5–10 min at 4 °C and aliquoted samples stored.

XSESS Study: Centrifugation in the operating theatre at 2000× *g* for 3 min, with supernatant aliquoted into six 100 µL cryovials and transferred for storage on dry ice.

### 2.3. Animal Ethics

Germinal vesicle (GV) oocytes were used in this study to investigate the redox status and RNA damage of oocytes to mimic the physiological time-point of greatest vulnerability of the oocytes to oxidative stress and RNA damage. GV oocytes collected from women for the process of IVF are used for their treatment and are therefore difficult to acquire for scientific research. A mouse model is commonly deemed a biologically and scientifically justified approach to overcoming ethical and practical difficulties with using human oocytes. The study met approval of the regional and University of Southampton ethics committees. All animal work has been carried out in adherence to the Animals Scientific Procedures Act of 1986 (ASPA) under project and personal licences granted by the Home Office (PIL number I02BABE9E).

### 2.4. Oocyte Collection and Preparation

Three- to four-week-old C57BL6 mice (Charles River, UK) were inbred and supplied by the Biomedical Research Facility (BRF), University of Southampton. Temperature was controlled at 18–23 °C and humidity at 40–60% in scantainers set to 12 h light and dark cycles. The mice received an ASPA approved diet which was available with water ad libitum. Enrichment was provided in their cages. They were injected via intra-peritoneal route with 10 IU pregnant mare serum gonadotrophin (PMSG, Centaur Services, UK) for follicle stimulation to increase the number of oocytes obtained from each mouse and reduce mouse numbers used for experiments. Forty-eight hours post-injection, mice were euthanised via cervical dislocation, and ovaries were dissected and placed in M2 medium containing milrinone (37 °C under mineral oil). Follicles were punctured using a 27-gauge needle, and oocytes were manually denuded using glass mouth pipettes on a heated microscope stage. To minimise oxidative stress, exposure to light was reduced, and all procedures were performed under dark conditions.

### 2.5. Gibson Cloning Protocol

The genetically encoded redox biosensor Grx1-roGFP2 was generated using Gibson assembly. SnapGENE software was used to design a suitable plasmid in silico which contained a pRN3 backbone and inserts for Grx1 and roGFP2, primers from Eurofins were then designed to span the Grx1-roGFP2 insert. A polymerase chain reaction (PCR) for the sections of the construct was performed with Q5 PCR buffer (New England Biolabs, Ipswich, MA, USA), oligonucleotides (Promega) and the Eurofins designed primers. Template DNA from ampicillin cultured E-Coli from Grx1-roGFP2 colony was added to the PCR reagents by dipping a sterilised pipette tip in a Grx1-roGFP2 positive E-Coli glycerol stock and agitating the pipette tip in the PCR reagents. PCR was carried out in a Bio-Rad T100 Thermo Cycler PCR machine (Bio Rad Laboratories, Hercules, CA, USA) by incubation at 98 °C for 30 s followed by 35 cycles of incubation at 98 °C for 10 s, then 70 °C for 20 s and 72 °C for 1 min 15 s. A final incubation at 72 °C was performed.

Presence of Grx1-roGFP2 DNA in the PCR product was confirmed with agarose gel electrophoresis. Concentration of the DNA was quantified by comparing integrated density of the representative band with the 1000bp band from of DNA ladder (known concentration 120 ng/uL) using ImageJ software. Grx1-roGFP DNA was then diluted to working concentration of 0.1 pmol with nuclease free water. DpnI digest was performed to degrade the methylated DNA. Pellet paint was then added to the PCR product and the product was cleaned with sodium acetate and 100% ethanol. The solution was centrifuged, and supernatant was removed. The pellet was washed in 70% ethanol and centrifugation repeated. Resulting pellet was then resuspended in nuclease free water.

The PCR-generated DNA fragments were then ligated into a pRN3 plasmid backbone with the Gibson reaction. Gibson reaction product was tested with gel electrophoresis against pRN3 plasmid backbone and Grx1-roGFP gene insert. Presence of new plasmid DNA from the individual PCR products was indicated on the gel with an additional band at the size of the combined band sizes of pRN3 and Grx1-roGFP2 genes.

Plasmids were transformed into *E. coli* by adding 5 μL of Gibson reaction plasmid product to 50 μL of New England Biolabs 5 alpha competent *E. coli*, flicked to mix and left on wet ice for 30 min. For DNA to be taken up by the bacteria, they were heat shock treated for 30 s at 42 °C on a heat block (Grant Industries, Elmwood Park, NJ, USA) and then placed back on ice. Super Optimal broth with catabolite repression (Sigma Aldrich, St. Louis, MI, USA) was added to the mixture and then placed in an incubator shaker at 37 °C and 250 rpm for 1 h. Transformed E-coli mixture was spread on an ampicillin agar plate in sterile conditions and incubated at 37 °C for 12–15 h. Successful recombinants were first verified by performing a PCR with GoTaq PCR reagents (Promega, Madison, WI, USA) followed by gel electrophoresis. They were then further verified by performing in vitro transcription template generation with Q5 HiFidelity PCR reagents (Invitrogen, Waltham, MA, USA) and two resulting DNA samples were sent for DNA sequencing (Eurofins Genomics, Louisville, KY, USA) with either a T3 primer or XFP Cterm 2 3′ primer added to the sample. Sequencing result demonstrated suitable generated Grx1-roGFP2 DNA and was used for in vitro transcription to RNA and the E-Coli colony was made into a glycerol stock by wiping a sterilised pipette tip into the colony from agar plate and stirring into LB broth with ampicillin (ampicillin 100 mg/mL concentration in LB broth in 1:1000 ratio) and placed in incubator shaker at 37 °C and 250 rpm for 12–15 h. The tube was then flicked, and contents were transferred into Eppendorf tube and centrifuged at 3000 rpm for 2 min. Two thirds of the supernatant was removed, and the pellet resuspended in the remaining third of solution. Glycerol was added in a 1:1 ratio and mixed by inverting the tube. This was stored in a cryovial at −80 °C.

The construct was transcribed in vitro using a mMessage T3 RNA polymerase kit (Ambion, Austin, TX, USA). Reagents were mixed and incubated at 37 °C in Bio Rad T100 Thermo-cycler PCR machine for 2 h. RNA was recovered by adding Lithium Chloride precipitation solution (Invitrogen) to the RNA product and freezing at −20 °C for 12–15 h. Product was then centrifuged at >13,000 rpm at 4 °C for 15 min. The pellet was cleaned by washing with 70% ethanol twice. The pellet was air dried at room temperature for 2 min and resuspended in 10 μL of nuclease free water. A small sample was tested for concentration of RNA on Nanodrop ND-1000 spectrophotometer (Thermo Fisher Scientific, Waltham, MA, USA). This concentration was used to dilute the remaining RNA sample to 500 ng/μL concentration suitable for microinjection and 0.5 μL volumes were stored for imminent use at −20 °C or for longer term use at −80 °C.

**Figure 1:**
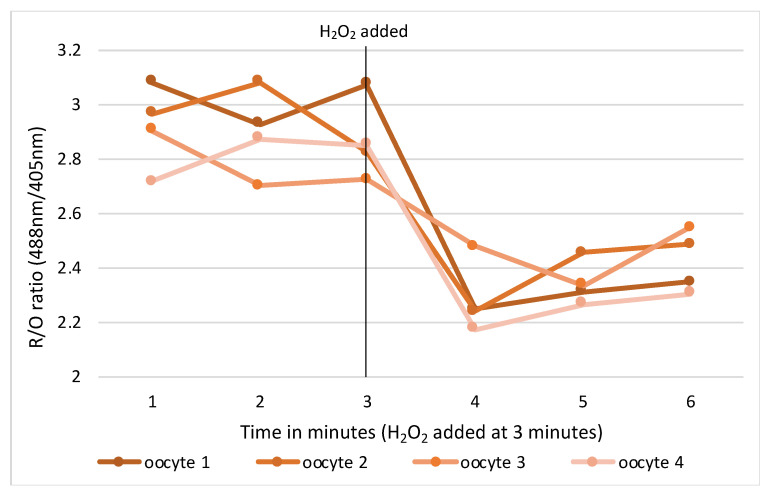
Change in intracellular redox status in oocytes pre and post oxidising agent (H_2_O_2_).

RNA microinjections were conducted in M2 media covered in mineral oil on the stage of an inverted TE300 microscope (Nikon, Tokyo, Japan), using a 37 °C heated chamber and micromanipulators (Narishige, Tokyo, Japan). RoGFP2 and H2B-mCherry were injected into GV mouse oocytes using timed pressure injections from a Picopump (World Precision Instruments, Sarasota, FL, USA) to achieve a size of 1–2% of the oocyte volume. Micro-injected oocytes were transferred back into a milrinone supplemented M2 media holding dish for 20 min at which time oocytes were assessed for viability and live cells selected for ongoing experimentation. Suitable oocytes were transferred in groups of 4–5 into drops of milrinone supplemented M2 media in wells of a flat-bottomed glass 96-well imaging plate. The oocytes were then imaged at 37 °C on a Leica SP8 confocal microscope fitted with hybrid detectors and a ×40/1.3NA plan apochromat oil immersion lens. Appropriate gain and power settings for 405 nm, 488 nm and 595 nm laser lines were selected with detection emission at 530 nm. Pinhole used was 2.5–5 and positions were marked for each oocyte focusing on the middle of the nucleus. Sequential imaging of channels was used to prevent bleedthrough from the multiple lasers to incorrect detection channels. Oocytes were imaged at 1-minute intervals for 10 min to establish a baseline, then either an oxidising (H_2_O_2_) or reducing (resveratrol) control agent for validation (Figure 1).

### 2.6. roGFP Experiments 

A total of four peritoneal fluid samples from women with stage III/IV endometriosis and five from controls were randomly selected. Oocytes microinjected with 0.1–0.3% (500 ng/µL) Grx1-roGFP2 RNA construct and H2B-mCherry and incubated in either 20% peritoneal fluid or control media at 37 °C. Redox status was assessed using confocal fluorescence microscopy:

Fluorescence imaging: Oocytes were sequentially excited at 405 nm (oxidised state) and 488 nm (reduced state), with emission collected at 530 nm.

Image analysis: Using ImageJ software (Version 1.53), hyperstack projections were created from z-stacks at the nuclear focal plane. Mean integrated density of fluorescence within a region of interest (plasma membrane) was quantified, with comparison of redox shifts across experimental conditions at baseline and at 4 h of incubation.

### 2.7. Immunofluorescence

Oocytes (*n* = 10–20 per group) were incubated in M2 media, H_2_O_2_, or peritoneal fluid (15–20%) for up to 16 h. Incubations were carried out in closed Eppendorf tubes under a layer of mineral oil. Eppendorfs were placed in small wells of a 37 °C heat block in a dark room. Oocytes were subsequently fixed with 37% formaldehyde, permeabilised with Triton X, blocked with Bovine Serum Albumin and 1% Tween 20, and stained with:

Primary antibody: Anti-8-OHG (RNA oxidation marker). The primary antibody of interest (anti-8-OHG) was mixed in blocking solution (1:100) and the oocytes were incubated in drops in the dark at 37 °C for 1 h under a coverslip. The oocytes were then washed in their groups through drops of 1% PVP/PBS.

Secondary antibody: Goat anti-rabbit Alexa Fluor. The above incubation and washing process was repeated for the secondary antibody (goat anti-rabbit) (1:1000 antibody in blocking solution).

Nuclear counterstain: DAPI or Hoechst. Staining of the DNA was then performed by incubating the groups of oocytes in drops of Hoechst or DAPI at 1:1000 concentration with blocking solution for 15 min in the dark at 37 °C under a coverslip. Oocytes were then washed in drops of 1% PVP/PBS.

Small drops of glycerol with citifluor antifade were placed onto an imaging dish and covered with mineral oil. The oocytes were then distributed to the drops in numbers of 6–7 according to their exposure group. Imaging was performed using confocal microscopy. ImageJ software was used for analysis, where mean fluorescence intensity (integrated density) within the cytoplasm was measured after subtracting nuclear fluorescence.

### 2.8. Statistical Analysis

All continuous data were expressed as means ± standard deviations (SDs). The normality of distributions was tested using Kolmogorov–Smirnov and Shapiro–Wilk tests.

For two-group comparisons, independent samples t-test or Wilcoxon signed-rank test was used, depending on normality.

For multiple comparisons, one-way analysis of variance (ANOVA) or Kruskal–Wallis test was performed, with Bonferroni correction for multiple testing.

Significance threshold: *p* < 0.05.

Software: Statistical analyses were conducted using GraphPad Prism v7 (GraphPad Software, San Diego, CA, USA) and SPSS v25 (IBM, London, UK).

## 3. Results

### 3.1. Human Samples

For redox experiments, samples from 9 of 62 women recruited to XSESS study were utilised. For an RNA damage experiment, samples from 12 women recruited to XSESS study were utilised. Univariate analysis of the main characteristics of all 62 women recruited to XSESS revealed that the control group and endometriosis group were similar by age, BMI and smoking status but that control group were more likely to have medical co-morbidities and more likely to have other gynaecological pathology noted at surgery. The endometriosis group were more likely to be nulliparous. 14 women were recruited to Peritoneal Fluid Biology in Health and Disease following the required ethical amendments. 7 samples from these women were utilised for an RNA damage experiment. Univariate analysis of the main characteristics of all 14 women recruited after amendments to Peritoneal Fluid Biology in Health and Disease revealed the control group and endometriosis group were similar by BMI, smoking status, and co-morbidity status but mean age in the control group was lower than the endometriosis group.

### 3.2. Redox Status in Oocytes Exposed to Peritoneal Fluid

**Figure 2:**
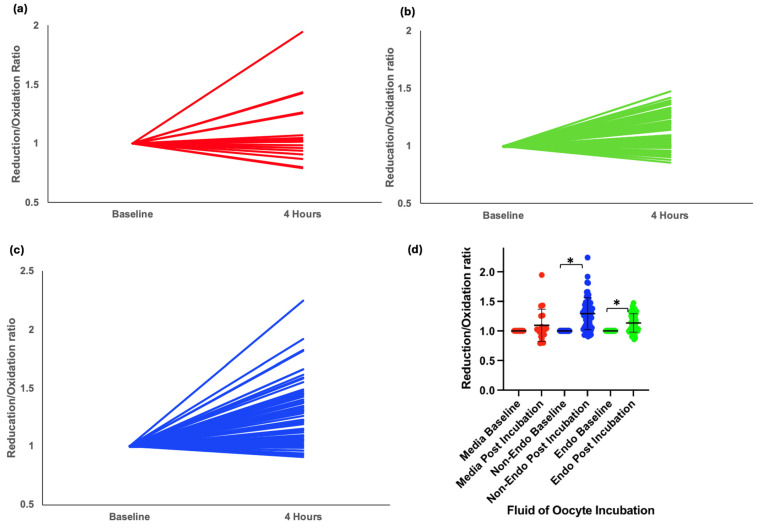
Intracellular redox status in mouse oocytes injected with Grx1-roGFP2 and incubated for 4 h in different conditions. (**a**) Graphical representation of change in redox status of oocytes incubated in control media. (**b**) Graphical representation of change in redox status of oocytes incubated in peritoneal fluid from women with endometriosis. (**c**) Graphical representation of change in redox status of oocytes incubated in peritoneal fluid from women without endometriosis. (**d**) Grouped scatter plot comparing the reduction/oxidation (r/o) ratios of oocytes across conditions. Data indicate a significant shift toward reduction in oocytes incubated in peritoneal fluid, with the most pronounced reduction observed in fluid from women without endometriosis (* *p* < 0.001).

A total of 141 oocytes were incubated in either media or 20% peritoneal fluid from nine women in the XSESS study (four with stage III/IV endometriosis and five controls with a normal pelvis). Data was normally distributed. Oocytes incubated in media alone showed no significant redox shift (mean change = 0.06, *p* = 0.26) (Figure 2a,d). However, exposure to peritoneal fluid resulted in a significant shift toward reduction in intracellular redox status, with the greatest reduction observed in oocytes exposed to fluid from women without endometriosis (mean change = 0.17, *p* < 0.001) (Figure 2c,d). In contrast, those exposed to fluid from women with endometriosis showed a smaller reduction (mean change = 0.07, *p* < 0.001) (Figure 2b,d).

**Figure 3:**
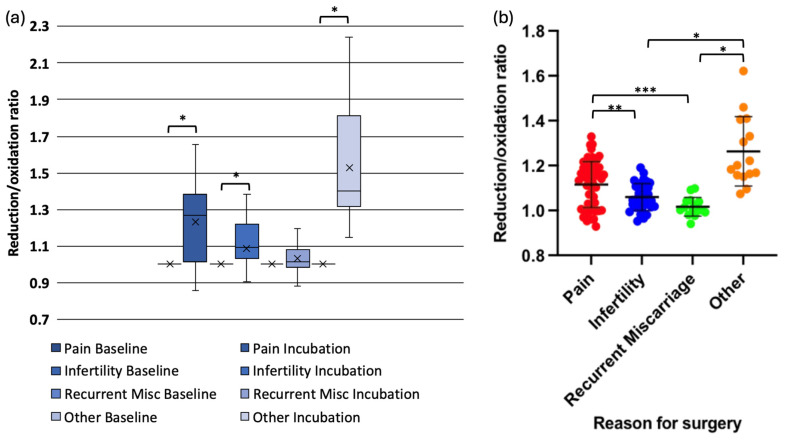
Redox status changes in mouse oocytes injected with Grx1-roGFP2 following incubation in peritoneal fluid from women with different clinical conditions. (**a**) Box-and-whisker plot showing the change in reduction/oxidation (r/o) ratio in oocytes after 4-hour incubation. (**b**) Grouped scatter plot comparing r/o ratios in oocytes exposed to peritoneal fluid from women with pelvic pain, infertility, recurrent miscarriage, (* *p* < 0.001, ** *p* < 0.003, *** *p* < 0.005).

When redox status was analysed by surgical indication, data in one subgroup (pain) was not normally distributed. Oocytes exposed to peritoneal fluid from women with pelvic pain (mean change = 0.13, *p* < 0.001), infertility (mean change = 0.05, *p* < 0.001), and asymptomatic individuals undergoing sterilisation (mean change = 0.29, *p* < 0.001) exhibited significant redox shifts. However, oocytes exposed to peritoneal fluid from women with recurrent miscarriages did not demonstrate a significant change (mean change = 0.02, *p* = 0.234) (Figure 3a,b).

Pairwise comparisons revealed that the reduction in r/o ratio was significantly lower in oocytes exposed to peritoneal fluid from women with infertility compared with those from women with pelvic pain (*p* = 0.003) or asymptomatic controls (*p* < 0.001). Similarly, oocytes exposed to peritoneal fluid from women with recurrent miscarriages exhibited significantly lower reduction compared with those from women with pelvic pain (*p* = 0.005) or asymptomatic individuals (*p* < 0.001). The reduction in redox status was significantly more in oocytes exposed to peritoneal fluid from asymptomatic women compared with those with pelvic pain (*p* = 0.001) (Figure 3a,b).

### 3.3. RNA Oxidative Damage in Oocytes

**Figure 4:**
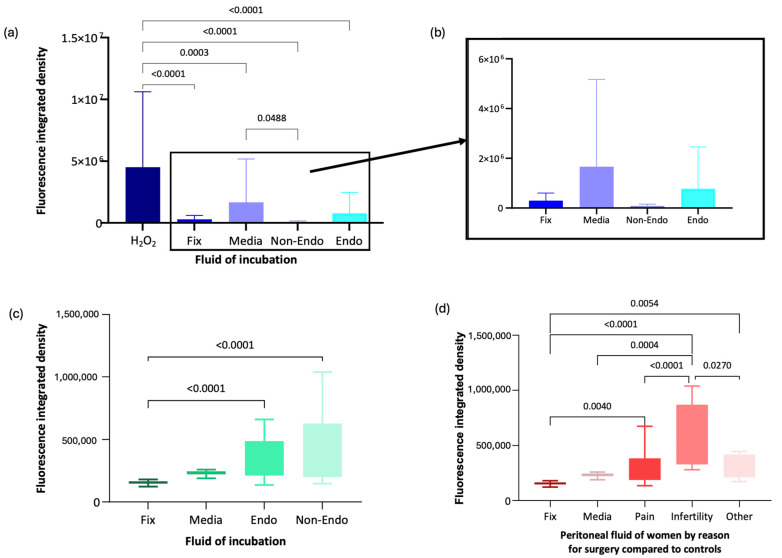
Immunofluorescence analysis of anti-8-OHG fluorescence intensity in oocytes under different conditions. (**a**) Box-and-whisker plot representation of anti-8-OHG fluorescence levels in oocytes from the Peritoneal Fluid Biology study. (**b**) Magnified box-and-whisker plot comparing fluorescence levels in fixed, media-incubated, and peritoneal fluid-exposed oocytes. (**c**) Box-and-whisker plot of anti-8-OHG fluorescence intensity in oocytes from the XSESS study. (**d**) Box-and-whisker plot comparing fluorescence levels by surgical indication in the XSESS study.

Immunofluorescence analysis using anti-8-OHG antibody was conducted on oocytes exposed to peritoneal fluid from women recruited to the Peritoneal Fluid Biology. Data was normally distributed. Mean fluorescence intensity (integrated density, pixels) in oocytes exposed to H_2_O_2_ (oxidising control) was significantly higher (mean = 4,514,263.9, SD = 6,102,798.3) compared with oocytes fixed immediately without incubation (mean = 295,469.1, SD = 304,826.9, *p* < 0.001), media-incubated oocytes (mean = 1,660,599.4, SD = 3,511,593, *p* < 0.001), and oocytes exposed to peritoneal fluid from women with endometriosis (mean = 777,589.3, SD = 1,681,373.5, *p* < 0.001) or without endometriosis (mean = 86,495.1, SD = 69,219.5, *p* < 0.001) (Figure 4a,b).

RNA damage was significantly lower in oocytes incubated in peritoneal fluid from women without endometriosis than in media alone (*p* = 0.049). However, there was no significant difference in RNA damage between oocytes exposed to peritoneal fluid from women with endometriosis and those from women without endometriosis (*p* = 0.721) or between oocytes exposed to peritoneal fluid from women with endometriosis and those fixed immediately (*p* = 0.93) (Figure 4a,b).

Following recruitment to the XSESS study, the anti-8-OHG antibody immunofluorescence methodology was applied to groups of 10–20 oocytes incubated for 16 h in media control and different peritoneal fluid samples (6 non endometriosis controls and 6 endometriosis cases) and compared to a group of oocytes fixed and imaged immediately or incubated in H_2_O_2_. Data in all groups were normally distributed apart from the media control group. Oocytes in the H_2_O_2_ group unfortunately did not survive incubation in the oxidising agent and were too damaged to analyse. Oocytes incubated in peritoneal fluid from women with (mean = 340,925.5, SD = 134,427.4) and without endometriosis (mean = 419,344.37, SD = 270,934.79) exhibited significantly higher RNA damage compared with oocytes fixed immediately (mean = 191,805, SD = 111,153.14, *p* < 0.001 for both comparisons) (Figure 4c). RNA damage in oocytes incubated in media was not significantly different from other incubation groups (Figure 4c).

When analysed by clinical subgroup, data was normally distributed except in the asymptomatic subgroup (women having surgery for sterilisation). Oocytes incubated in peritoneal fluid from women undergoing surgery for infertility exhibited significantly more RNA oxidative damage, with a 1.87-fold increase compared to asymptomatic individuals (mean = 571,708.22, SD = 255,954.17, *p* = 0.027) and a 1.78-fold increase compared to those with pelvic pain (mean = 321,924.99, SD = 171,072.10, *p* < 0.001) (Figure 4d). Oocytes exposed to peritoneal fluid from women with pelvic pain showed only a 1.05-fold increase in RNA oxidative damage compared to asymptomatic individuals. Oocytes fixed immediately had significantly lower RNA damage than those incubated in peritoneal fluid from women with infertility (*p* = 0.004), pelvic pain (*p* = 0.005), or asymptomatic individuals (*p* < 0.001) (Figure 4d).

## 4. Discussion

This study investigated the impact of peritoneal fluid from women with and without endometriosis on mouse oocytes’ intracellular redox status and RNA integrity. Our redox experiments, using microinjection of the Grx1-roGFP2 biosensor, revealed that oocytes exposed to fluid from women without endometriosis exhibited a more significant reduction (mean change in r/o ratio 0.17, *p* < 0.001) than those exposed to fluid from women with endometriosis (mean change 0.07, *p* < 0.001). The peritoneal fluid of women with endometriosis had a more oxidative effect. Furthermore, subgroup analyses showed that peritoneal fluid from women with infertility and recurrent miscarriage induced significantly less reduction than that from women with pelvic pain or asymptomatic individuals, suggesting that a less reducing intracellular environment, possibly indicative of higher ROS or lower antioxidant capacity, may adversely affect oocyte quality. These findings are consistent with the concept that ROS and antioxidants contribute to the oocyte maturation process and overall quality.

Parallel immunofluorescence studies employing an anti-8-OHG antibody to assess RNA oxidative damage produced mixed outcomes. In one cohort, oocytes incubated in peritoneal fluid from women without endometriosis displayed significantly lower RNA damage than those in media. However, comparisons between oocytes exposed to peritoneal fluid from women with versus without endometriosis did not yield statistically significant differences, and a separate cohort revealed increased RNA damage in oocytes incubated in fluid from women undergoing surgery for infertility. These inconsistencies may reflect limitations in the sensitivity of the immunofluorescence method to detect RNA damage at the physiological level or differences in sample processing, underscoring that the redox buffering capacity of peritoneal fluid remains poorly characterised.

Redox experiments also revealed that oocytes incubated in peritoneal fluid significantly shifted toward reduction compared to those in media alone. The observation that peritoneal fluid from women results in more significant reduction compared to more redox-stable media may seem counterintuitive, it may be suggested that a more reduced redox state is protective/beneficial to the oocyte. The expectation of a more oxidative milieu in the peritoneal fluid of cases has not been observed, but there is less reduction which may point to higher ROS or lower antioxidant capacity. In addition, inherent variations in redox buffering capacity and methodological factors, such as the sensitivity and dynamics of the Grx1-roGFP2 biosensor, could contribute to these results. The complex nature of oocyte redox regulation further complicates interpretation [17].

In terms of novelty, our study represents a significant advancement over previous research. By integrating the use of a genetically encoded redox biosensor (Grx1-roGFP2) with immunofluorescence detection of RNA oxidative damage (anti-8-OHG), we provide a direct and dynamic assessment of how peritoneal fluid influences oocyte quality. Moreover, the comparison of peritoneal fluids from diverse clinical groups, including endometriosis, infertility, pelvic pain, recurrent miscarriage, and asymptomatic individuals, offers novel insights into the heterogeneity of the peritoneal microenvironment and its potential impact on reproductive outcomes. This multifaceted approach addresses a critical gap in our understanding of the redox buffering capacity of peritoneal fluid and its role in oocyte health.

It is important to note that significant ethical and practical barriers limit the use of human oocytes in research; thus, mouse oocytes were employed as a surrogate model. Using mouse oocytes may limit direct extrapolation to human fertility. Future studies using human-derived gametes will be critical to validate these findings. Other limitations noted include the sensitivity of immunofluorescence with anti-8-OHG antibody to physiological levels of damage to RNA as discussed. The level of RNA damage in oocytes exposed to peritoneal fluid may be too subtle for the technique of immunofluorescence to accurately measure. In small human studies, some differences were found between the groups either in age or in presence of co-morbidities (Peritoneal Fluid Biology and XSESS, respectively). This may be due to inherent differences between women with and without endometriosis or may be an impact of a small scale human study. Although significant findings have been demonstrated, the data is limited by sample size. A wide spread of data across some analysis groups was noted. This study indicates suitable techniques have been identified with microinjection of the biosensor Grx1-roGFP2 and immunofluorescence for damaged RNA bases. Larger scale experiments with a greater number and range of human peritoneal fluid samples and bigger oocyte groups would now be beneficial in understanding the impact further and performing more robust sub-group analyses.

In conclusion, while peritoneal fluid provides a more reducing environment than standard media, which may protect oocyte integrity, alterations in this redox balance, particularly in pathological states, could compromise oocyte quality. Further studies with larger, more uniform sample sets are warranted to elucidate these complex interactions and better to define the redox buffering capacity of peritoneal fluid.

## Data Availability

The original data presented in the study are openly available in University of Southampton Institutional Repository at https://doi.org/10.5258/SOTON/D3025.
